# Impact of Hydrocodone Rescheduling on Pharmaceutical Pain Management in End-of-Life Lung Cancer Patients

**DOI:** 10.1177/08258597251398132

**Published:** 2025-11-24

**Authors:** Chan Shen, Mohammad Ikram, Shouhao Zhou, Roger Klein, James Douglas Thornton, Douglas Leslie

**Affiliations:** 1 Department of Surgery, 8082 The Pennsylvania State University, College of Medicine, Hershey, PA, USA; 2 Department of Public Health Sciences, The Pennsylvania State University, College of Medicine, Hershey, PA, USA; 3 Penn State Cancer Institute, Hershey, PA, USA; 4 Department of Economics, 5970 Rutgers University, New Brunswick, NJ, USA; 5 Department of Pharmaceutical Health Outcomes and Policy, 15507 University of Houston College of Pharmacy, Houston, TX, USA; 6 Prescription Drug Misuse Education and Research (PREMIER) Center, University of Houston College of Pharmacy, Houston, TX, USA

**Keywords:** pain management, lung cancer, end of life, opioid and nonopioid pharmacotherapy, hydrocodone rescheduling, SEER-Medicare data

## Abstract

**Objective:**

Lung cancer is the leading cause of cancer mortality in the USA, and terminal patients endure severe pain. In October 2014, hydrocodone was rescheduled from Schedule III to Schedule II to curb misuse, yet its specific impact on end-of-life lung cancer pain management remains unclear. The objective of this study is to evaluate the impact of hydrocodone rescheduling on pharmaceutical pain management among end-of-life lung cancer patients in the USA.

**Methods:**

We conducted a retrospective study using SEER-Medicare data including 24,804 patients aged ≥66 with stage IV lung cancer who died between 2011 and 2019. We examined hydrocodone, opioid, NSAID, and antidepressant use in the last three months of life and used multivariable logistic and OLS regression to assess the effects of hydrocodone rescheduling and time trends, adjusting for demographic and clinical factors.

**Results:**

Hydrocodone was used by 47.5% of patients and any opioids by 75.4%. Although the hydrocodone rescheduling showed a nonsignificant immediate reduction in use (AOR = 0.92, *p =* .0795) and dosages (estimate = -1.78 MME, *p =* .358), significant declining trends were observed over time for hydrocodone (AOR = 0.92 per 12 months, *p <* .001; −1.15 MME per 12 months, *p =* .003) and overall opioids (AOR = 0.94 per 12 months, *p <* .001; −2.44 MME per 12 months, *p =* .002). NSAIDs and antidepressants use remained stable.

**Conclusion:**

Our study is the first to examine hydrocodone rescheduling's impact on pain management in end-of-life lung cancer care. While no immediate significant changes were observed, the overall decline in opioid use over time reflects broader clinical and regulatory shifts, highlighting the need for balanced, multimodal pain management strategies.

## Introduction

Lung cancer is the leading cause of cancer mortality in the USA, causing 125,070 deaths or 20.4% of all cancer deaths in 2024.^1^ Beyond its high mortality rate, lung cancer is characterized by severe symptomatology, with pain being one of the most debilitating and pervasive issues experienced by patients. The etiology of pain in lung cancer is multifactorial, arising from tumor invasion, treatment-related side effects, and associated comorbid conditions.^2,3^ The terminal phase of lung cancer is often marked by an escalation in pain intensity due to rapid disease progression and the cumulative burden of treatment side effects.^4,5^ During end-of-life care, patients not only endure severe physical discomfort but also face significant emotional and psychological challenges,^6,7^ making effective pain management even more critical.

Opioids play a critical role in managing the severe pain experienced by lung cancer patients. They are widely regarded as the cornerstone of pain management in this population.^8^ Their efficacy in managing moderate to severe pain has led to their routine use for alleviating the multifaceted pain experienced during the end-of-life stage, when the primary focus is on symptom relief and enhancing overall well-being. Optimal opioid use is essential for enhancing quality of life in a patient's final days. This underscores the need to thoroughly understand opioid utilization patterns and the myriad factors influencing their prescription in end-of-life care for lung cancer patients.

Recent studies have begun to shed light on a concerning trend: a decline in opioid use among cancer patients during end-of-life care. Several investigations have documented reduced opioid prescription rates, suggesting that despite their critical role in managing severe pain, these medications are being prescribed less frequently in terminal care settings.^9,10^ For example, a study of 270,632 Medicare beneficiaries with poor prognosis cancers (including lung cancers) who died between 2007 and 2017 found the proportion of patients filling ≥1 opioid prescription near end-of-life decreased from 42.0% to 35.5%.^9^ The study also reported that Long-acting opioid prescriptions declined by 36.5%, from 18.1% to 11.5% while the total amount of opioids prescribed per decedent near end-of-life decreased by 38.0%.^9^ Concurrent with the decline in opioid access the study reported that pain-related emergency department visits increased by 50.8%, from 13.2% to 19.9% of patients.^9^

This decline in opioid prescribing may be attributed to factors such as evolving clinical guidelines, increased regulatory scrutiny, concerns over opioid misuse, and a heightened awareness of potential adverse effects. Policy change is one of these important factors. In particular, hydrocodone was reclassified from Schedule III to Schedule II in October 2014, marking a pivotal policy shift aimed at curbing opioid misuse. This rescheduling imposed stricter prescribing and dispensing controls, and many studies in the literature have shown that hydrocodone prescribing and dispensing decreased significantly after this policy change.^11–13^ However, the specific effects of rescheduling on end-of-life care for lung cancer patients remain largely unexplored, and a comprehensive study including not only hydrocodone but any opioids and other pharmaceutical pain management options such as NSAIDs and antidepressant is warranted. This study seeks to address this knowledge gap by evaluating the impact of hydrocodone rescheduling on all the above pharmaceutical pain management modalities among end-of-life lung cancer patients, providing much-needed insights into its implications for palliative care and pain management in this vulnerable population.

## Methods

### Data Source

The data source for this study is the Surveillance, Epidemiology, and End Results (SEER) data linked with Medicare claims for patients aged 65 years and older. Supported by the National Cancer Institute (NCI), SEER is a widely recognized source for population-based cancer research, covering more than 35% of the US population.^14^ It provides comprehensive data on patient demographics, primary tumor sites, cancer stage at diagnosis, date of diagnosis, and survival outcomes including date of death for deceased individuals. The linkage with Medicare claims enhances the dataset by incorporating detailed information on healthcare utilization before and after cancer diagnosis for Medicare beneficiaries, including claims from Parts A, B, and D. Since 2007, Medicare Part D claims have included detailed records of pharmaceutical prescriptions, encompassing prescription opioids and other medications.

### Study Cohort

The study cohort consisted of Medicare beneficiaries aged ≥66 years who were diagnosed with AJCC Sixth Edition Stage IV lung cancer and died between 1 January 2011, and 31 December 2019, excluding cases diagnosed at autopsy. This timeframe captures the 2014 policy change regarding hydrocodone rescheduling while excluding the COVID-19 pandemic period beginning in 2020. End of life was defined as the last three months before death, and patients who died within three months of diagnosis were excluded. We included only patients who had continuous enrollment in Medicare Parts A, B, and D, with no enrollment in a health maintenance organization (HMO) for the 12 months preceding death. This criterion ensured complete records for assessing comorbidities and cancer treatment during the study period.

### Pharmacotherapy for Pain Management at the End of Life

The use of hydrocodone and other opioids was identified based on generic drug names listed in Medicare Part D claims. Opioid use was confirmed if a patient's Part D claims included any of the following medications: hydrocodone, codeine, dihydrocodeine, fentanyl, hydromorphone, buprenorphine, methadone, levorphanol, meperidine, morphine, opium, oxycodone, oxymorphone, tapentadol, or tramadol. Similarly, the use of nonsteroidal anti-inflammatory drugs (NSAIDs) and antidepressants was determined based on their generic names.^15^

Opioid doses were converted to Morphine Milligram Equivalents (MME) using the conversion algorithm endorsed by the Centers for Disease Control and Prevention (CDC).^16^ The average daily MME was calculated by incorporating the days’ supply and active opioid prescription periods.

### Patient Characteristics

The demographic characteristics considered included age at diagnosis and race/ethnicity, categorized as African American, Hispanic, non-Hispanic White, and Other. Medicaid dual eligibility (yes/no) served as a proxy for socioeconomic status. Overall health status was assessed using the Charlson comorbidity score, derived from ICD-9, ICD-10, and CPT/HCPCS codes recorded in the insurance claims data during the 12 months before death.^17^ Further, we incorporated a specific indicator for depression, given its significant influence on pain perception and management; cancer treatments—including radiation, chemotherapy, and surgery—were identified also using codes in insurance claims, as these treatments can have a substantial impact on pain management strategies.

### Statistical Analysis

We provide the descriptive statistics, including frequencies and percentages, to summarize study sample characteristics. Monthly time trends from 2011 to 2019 were visualized to illustrate the percentage of patients using hydrocodone, any opioids, NSAIDs, and antidepressants during the last three months of life. Additionally, monthly trends in average daily MME for hydrocodone and any opioids were depicted over the same period.

We conducted multivariable logistic regression analyses to assess factors associated with hydrocodone use. These models included two key variables to evaluate both the policy impact and the overall temporal trend: a binary indicator for the post-policy period (i.e., after October 2014), and a continuous time variable representing monthly trends. For interpretability, time trend estimates are reported as odds ratios standardized to a 12-month period.

The models were adjusted for patient characteristics, including age, race/ethnicity, Medicaid dual eligibility, depression, Charlson comorbidity index, and cancer treatments. Similar multivariable logistic regression models were applied to examine the use of any opioids, NSAIDs, and antidepressants. Results were reported as adjusted odds ratios (AORs) with 95% confidence intervals (CIs) and corresponding *p*-values.

Ordinary least squares (OLS) regression models were used to assess average daily MME for hydrocodone and for all nonhydrocodone opioids. Each model included a binary indicator for the post-policy period (i.e., after October 2014) to estimate the immediate effect of hydrocodone rescheduling, as well as a continuous monthly time variable to capture temporal trends. For interpretability, time trend estimates were standardized to reflect the average change in MME per 12-month period. All models were adjusted for patient demographic and clinical characteristics, and results are presented as parameter estimates with corresponding standard errors (SEs) and *p*-values.

All statistical analyses were performed using SAS version 9.4 (SAS Institute, Cary, NC, USA), with two-sided tests conducted for all comparisons. This retrospective observational study was approved by the Institutional Review Board.

## Results

Table 1 provides a detailed description of the study sample, which includes 24,804 patients with stage IV lung cancer who were diagnosed and deceased between 2008 and 2019. The age distribution is fairly even, with 23.0% aged 66–69 years, 27.7% aged 70–74, 22.2% aged 75–79, and 27.1% aged 80 years or older. In terms of race and ethnicity, the majority of patients are Non-Hispanic White (78.0%), followed by African American (8.9%), Hispanic (5.2%), and other races (7.9%). In terms of pain management medication use, 47.5% of patients received hydrocodone, 75.4% received prescription opioids, 24.1% used NSAIDs, and 21.6% were on antidepressants.

**Table 1.** Sample Description of the Study.

	Total (*N* = 24,804)
Age	
66–69	5314 (23.0%)
70–74	6416 (27.7%)
75–79	5139 (22.2%)
≥80	6282 (27.1%)
Race/ethnicity	
Non-Hispanic White	19335 (78.0%)
African American	2218 (8.9%)
Hispanic	1298 (5.2%)
Other race	1953 (7.9%)
Sex	
Male	11618 (46.8%)
Female	13186 (53.2%)
Dual eligibility	
Yes	8107 (32.7%)
No	16697 (67.3%)
Depression	
Yes	7446 (30.0%)
No	17358 (70.0%)
Charlson comorbidity	
0	1343 (6.5%)
1	3439 (16.7%)
2	4554 (22.2%)
3 or more	11197 (54.5%)
Radiation therapy	
Yes	12542 (50.6%)
No	12262 (49.4%)
Chemotherapy	
Yes	15083 (60.8%)
No	9721 (39.2%)
Surgery	
Yes	2729 (11.0%)
No	22075 (89.0%)
Hydrocodone	
Yes	11780 (47.5%)
No	13024 (52.5%)
Any opioids	
Yes	18694 (75.4%)
No	6110 (24.6%)
NSAIDs	
Yes	5981 (24.1%)
No	18823 (75.9%)
Antidepressants	
Yes	5364 (21.6%)
No	19440 (78.4%)

Table 2 presents the multivariable logistic regression results examining the association of hydrocodone, any opioids, NSAIDs, and antidepressants use with the hydrocodone rescheduling and time trends over a 12-month period, after adjusting for age, race/ethnicity, sex, dual eligibility, depression, Charlson comorbidity score, and cancer treatments. For hydrocodone use, the policy change was associated with a nonsignificant reduction in odds (AOR = 0.92; 95% CI: 0.83-1.01; *p =* .0795), while a significant decreasing trend was observed over time (AOR = 0.92; 95% CI: 0.91-0.94; *p <* .001). Similarly, the policy change did not significantly affect any opioids use (AOR = 0.98; 95% CI: 0.88-1.10; *p =* .707), but there was a significant 6% reduction in odds per 12 months (AOR = 0.94; 95% CI: 0.92‒0.96; *p <* .001). In contrast, NSAIDs use showed no significant association with the policy change (AOR = 0.95; 95% CI: 0.85‒1.06; *p =* .3771) or with the time trend (AOR = 1.01; 95% CI: 0.98‒1.03; *p =* .5429). Similarly, neither the policy change (AOR = 1.05; 95% CI: 0.93‒1.19; *p =* .4057) nor the time trend (AOR = 0.98; 95% CI: 0.96‒1.01; *p =* .17) was significantly associated with changes in antidepressant use.

**Table 2.** Multivariable Logistic Regression Results for Hydrocodone, Any Opioids, NSAIDs, and Antidepressants, Including Immediate Effect of Hydrocodone Rescheduling (Post-Policy Indicator) and Time Trend (Odds Ratio per 12-Month Period).

Variables	AOR	95% CI	*p*
Hydrocodone use			
Policy change			
Post policy change	0.92	[0.83, 1.01]	.0795
Before policy change (reference)			
Time trend			
In 12 months	0.92	[0.91, 0.94]	<.001
Any opioids use			
Policy change			
Post policy change	0.98	[0.88, 1.10]	.707
Before policy change (reference)			
Time trend			
In 12 months	0.94	[0.92, 0.96]	<.001
NSAIDs use			
Policy change			
Post policy change	0.95	[0.85, 1.06]	.3771
Before policy change (reference)			
Time trend			
In 12 months	1.01	[0.98, 1.03]	.5429
Antidepressants use			
Policy change			
Post policy change	1.05	[0.93, 1.19]	.4057
Before policy change (reference)			
Time trend			
In 12 months	0.98	[0.96, 1.01]	.17

Note: The models were adjusted for age groups, race/ethnicity, sex, dual eligibility, presence of clinically diagnosed depression, Charlson comorbidity score, and cancer treatments (including radiation, chemotherapy, and surgery). The binary “post policy change” indicator captures the immediate effect associated with hydrocodone rescheduling (October 2014). The continuous “time trend” variable reflects monthly changes and is standardized to represent the adjusted odds ratio per 12-month period for ease of interpretation.

Table 3 displays the multivariable ordinary least squares regression results for hydrocodone MME and any opioids MME, adjusted for age, race/ethnicity, sex, dual eligibility, clinically diagnosed depression, Charlson comorbidity score, and cancer treatments. For hydrocodone MME, the policy change was associated with a nonsignificant reduction (estimate = ‒1.78, SE = 1.93, *p =* .358), whereas the time trend revealed a statistically significant decrease of 1.15 MME per 12-month period (SE = 0.393, *p =* .003). Similarly, for any opioids MME, the policy change effect was not significant (estimate = ‒2.18, SE = 3.26, *p =* .503), but a significant decline of 2.44 MME per 12 months was observed (SE = 0.652, *p =* .002). These findings suggest that although the hydrocodone rescheduling policy did not have a significant impact on opioid dosage levels, there is a notable and statistically significant downward trend in opioid dosages over time.

**Table 3.** Multivariable Ordinary Least Squares Regression Results for Hydrocodone and Any Opioids Average Daily MME, Including Immediate Policy Effect and Time Trend (Per 12-Month Period).

Variables	Estimate	SE	*p*
Hydrocodone use			
Policy change			
Post policy change	−1.777	1.932	.358
Pre policy change (reference)			
Time trend			
in 12 months	−1.146	0.393	.003
Any opioids use			
Policy change			
Post policy change	−2.178	3.256	.503
Before policy change (reference)			
Time trend			
in 12 months	−2.436	0.652	.002

Note: The models were adjusted for age groups, race/ethnicity, sex, dual eligibility, presence of clinically diagnosed depression, Charlson comorbidity score, and cancer treatments (including radiation, chemotherapy, and surgery). The “post policy change” variable represents the immediate level shift following hydrocodone rescheduling (October 2014). The “time trend” variable reflects month-to-month changes and is scaled to show the estimated change in average daily MME per 12-month period for interpretability.

**Figure 1.**
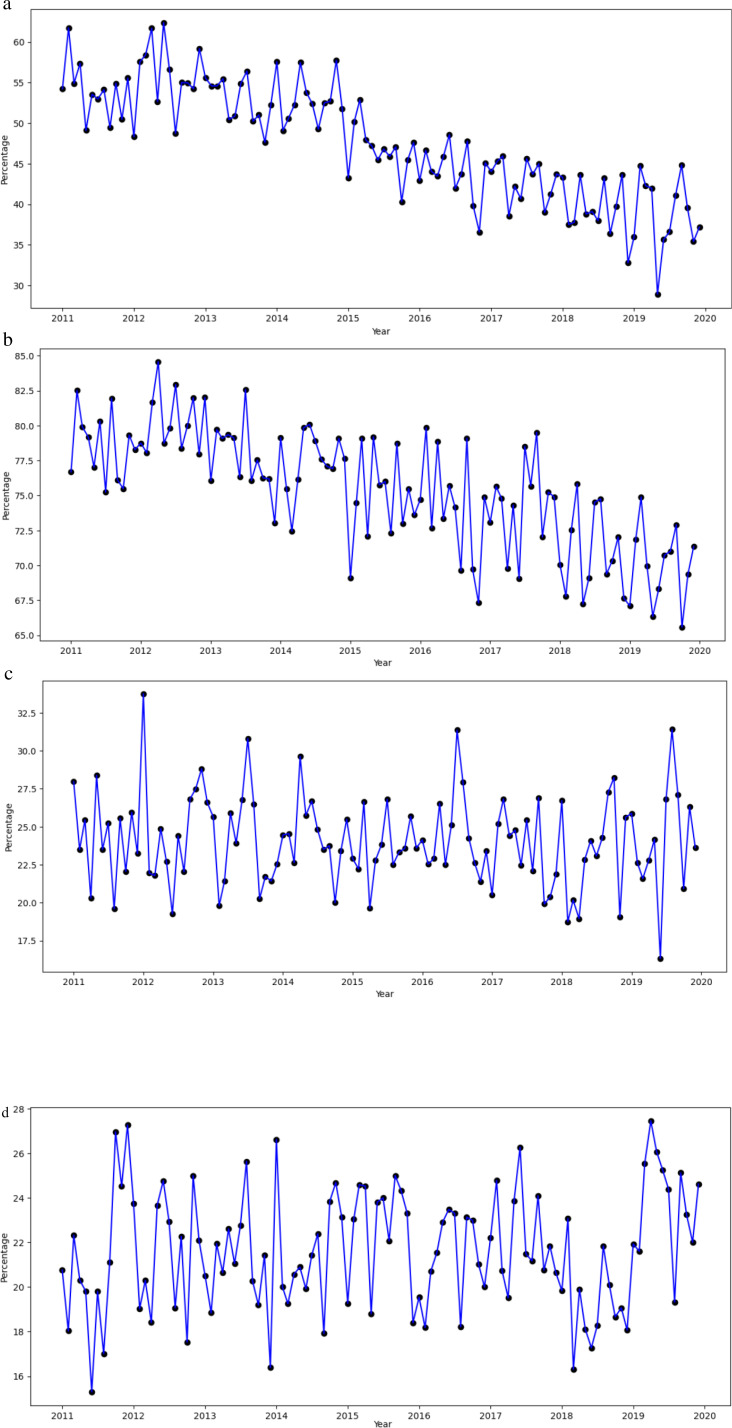
Monthly Percentage of Patients Using Hydrocodone, Any Opioids, NSAIDs, and Antidepressants. (a) Hydrocodone; (b): Any Opioids; (c): NSAIDs; (d): Antidepressants.

Figure 1 presents the monthly percentage of patients using hydrocodone, any opioids, NSAIDs, and antidepressants from 2011 to 2020. Figure 1(a) and (b) for hydrocodone use and any opioid use reveal a generally declining trend throughout the study period. Although some fluctuations are evident, the overall trajectory moves steadily downward, aligning with the logistic regression findings presented in Table 2, which indicate a significant decrease in hydrocodone and any opioid use over time. In contrast, Figure 1(c) and (d) for NSAIDs and antidepressants use show relatively stable utilization levels over the entire period, with only minor fluctuations. This lack of a clear upward or downward trend is in line with the multivariable logistic regression results in Table 2, which indicate no significant association of NSAIDs and antidepressant use with the policy change or the continuous time trend.

**Figure 2.**
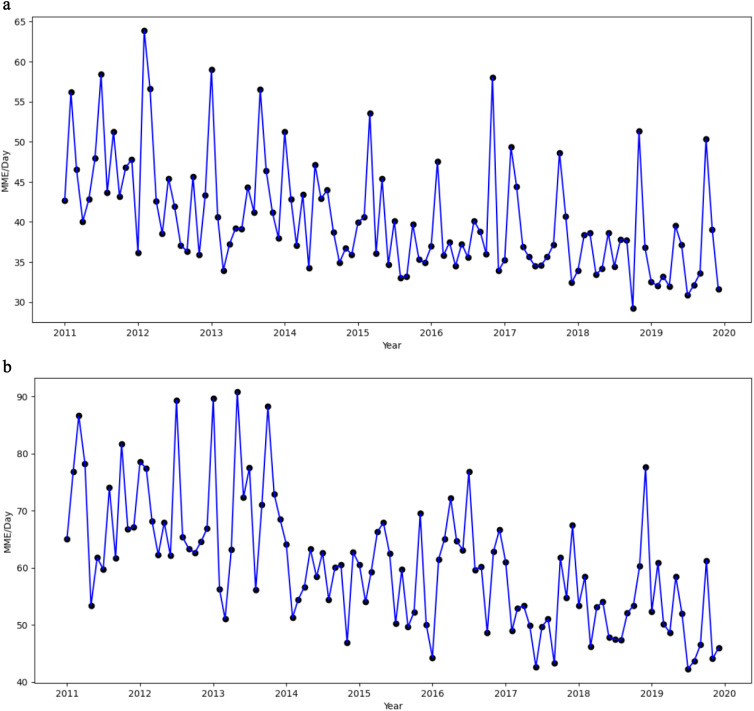
Monthly Trends of Opioid Charlson comorbidity (MME) per Day for Hydrocodone and Any Opioids. (a) Monthly Trend in Average Daily MME for Hydrocodone. (b) Monthly Trend in Average Daily MME for Any Opioid.

Figure 2 shows the monthly average MME for hydrocodone and any opioids from 2011 to 2020, highlighting a generally declining pattern over the study period, with sizable fluctuations. This downward trajectory closely aligns with the ordinary least squares regression findings (Table 3), which indicate a steady negative time trend in opioid MME.

## Discussion

This paper investigated the impact of the rescheduling of hydrocodone from a Schedule III to a Schedule II controlled substance on pain management specifically among end-of-life lung cancer patients. We analyzed the use of hydrocodone, nonhydrocodone opioids, NSAIDs, and antidepressants before and after the rescheduling. Additionally, we examined the effects on opioid dosage by evaluating trends in daily MME. To our knowledge, this is the first study to report these findings in this vulnerable patient group. Our analysis indicates that the 2014 hydrocodone rescheduling did not produce a statistically significant immediate shift in hydrocodone use or dosages among end-of-life lung cancer patients. Instead, a consistent downward trend emerged over the study period for both hydrocodone and any opioids, suggesting a broader, continuous decline in opioid prescribing rather than a discrete change coinciding with the policy shift in end-of-life lung cancer patients. This sustained decline likely reflects the cumulative influence of multiple national initiatives that reshaped opioid prescribing during the study period, most notably, the CDC's 2016 Guideline for Prescribing Opioids for Chronic Pain.^16^ Although the guideline explicitly excluded patients receiving active cancer treatment, palliative care, or end-of-life care, several studies have documented accelerated declines in opioid prescribing after 2016, suggesting unintended spillover effects into populations beyond the guideline's intended scope.^18–22^ As such, the observed trends in our study may reflect not only the impact of hydrocodone rescheduling, but also broader changes in the policy and regulatory environment influencing clinician behavior during this period.

These findings contrast with many studies in the literature that have reported significant declines in hydrocodone dispensing and compensatory increases in the prescribing of other opioids following hydrocodone rescheduling. For instance, a recent literature review found that while hydrocodone rescheduling was associated with reductions in the prescribing and use of hydrocodone-containing products, it also led to increased prescribing and use of other opioids.^23^ Similarly, another nationwide study by the FDA published in 2023 showed that hydrocodone rescheduling accelerated declines in hydrocodone dispensing, which were only partially offset by smaller increases in the dispensing of other opioids.^24^ The absence of a significant immediate effect in our study cohort may be attributed to factors unique to the end-of-life care context. First, providers caring for terminal lung cancer patients are often already exercising heightened caution with opioid prescriptions, aiming to balance effective pain relief with the minimization of adverse effects,^25–28^ thereby reducing the potential for dramatic shifts following a policy change. Additionally, competing clinical priorities in palliative settings—such as the urgent need to manage multifaceted symptoms and ensure patient comfort—may render the influence of a single regulatory intervention less pronounced.^28,29^ It is also possible that concurrent changes in clinical guidelines and broader regulatory pressures, which have been increasingly focused on opioid safety, contributed to a gradual reduction in hydrocodone use over time. Finally, heightened opioid awareness among both prescribers and patients may have led to a proactive shift away from hydrocodone even before its official rescheduling,^30,31^ reflecting an evolving practice environment that prioritized alternative pain management strategies well in advance of policy implementation.

Our findings are consistent with national trends showing reduced opioid dispensing among patients with poor-prognosis near the end of life, raising concerns about undertreatment and avoidable acute care utilization.^9^ For example, Enzinger et al. reported marked declines in MME prescribed from 2007 to 2017, accompanied by increasing barriers to opioid access.^9^ Notably, the proportion of patients experiencing pain-related emergency department visits rose by 50.8% over this period. In a separate national survey of 371 hospice facilities, 28% reported hydromorphone shortages, highlighting system-level disruptions in opioid availability.^32^ Although not all studies focus exclusively on end-of-life care, the broader literature links reduced opioid access with increased emergency department use and inadequate pain control—patterns that may reflect unmet needs in palliative settings and warrant further investigation.

Although our models adjusted for patient-level factors including race/ethnicity, sex, and dual eligibility as a proxy for socioeconomic status, we did not prespecify subgroup analyses. However, prior work shows persistent disparities in cancer-pain opioid access and dosing for racially and ethnically minoritized patients, including near the end of life.^33–35^ Future research is warranted to examine whether the effects of hydrocodone rescheduling and broader shifts in opioid availability have disproportionately impacted certain subpopulations, potentially exacerbating inequities in symptom control during the terminal phase of illness.

In contrast, our study found that NSAIDs and antidepressants use remained relatively stable over time, with no clear compensatory increase or decrease following hydrocodone rescheduling. These findings have important clinical implications for end-of-life pain management. In terminally ill patients, balanced pain control is essential to ensure comfort and maintain quality of life, and any unwarranted reduction in opioid prescribing risks leaving patients with inadequately managed pain.^36^ While opioid use declined over time in our cohort, the unchanged use patterns of NSAIDs and antidepressants suggest that nonopioid modalities are not being used as compensatory alternatives—possibly reflecting both clinical limitations and prescriber caution. Currently, there is a lack of robust evidence to either support or refute the use of NSAIDs alone or alongside opioids for cancer pain management, and only very low-quality evidence suggests that some patients with moderate or severe cancer pain may obtain substantial levels of benefit from NSAIDs.^37^ Moreover, although depression is highly prevalent in palliative care settings,^38^ studies have shown that patients are often undertreated with antidepressants.^39^ These findings underscore the need for further research into comprehensive, multimodal approaches to pain management that combine opioid and nonopioid therapies tailored to individual patient needs, ensuring that efforts to reduce opioid misuse do not inadvertently compromise effective symptom relief in end-of-life care.

Our study has several limitations. First, as a retrospective observational study, it is subject to potential confounding factors that may not be fully accounted for despite including many factors. The reliance on administrative claims data, while providing a large dataset, may not capture all the nuances of clinical decision-making and patient-provider interactions that influence prescribing patterns. A key limitation of this study is the absence of direct patient-reported outcomes, including pain severity, symptom control, functional status, satisfaction with care, and overall quality of life. While SEER–Medicare offers valuable insight into prescribing patterns, it does not capture validated measures of patients’ subjective experiences or clinical assessments of pain relief adequacy. Consequently, we are unable to determine how the observed changes in hydrocodone and other analgesic use translated into patients’ comfort or well-being during end-of-life care. This limits our ability to assess the clinical appropriateness of pain management practices during this period. Claims-based measures of depression likely underestimate the true prevalence of this condition.^40,41^ It is important to note that our analysis was restricted to prescription NSAIDs recorded in Medicare Part D claims and therefore does not account for over-the-counter NSAID use, which is not reimbursed or documented in this dataset. Consequently, overall NSAID utilization is likely underestimated, and the observed stability in prescription NSAID use may not fully reflect patients’ total exposure to these medications. Future research should aim to integrate nuanced clinical assessments or patient-reported outcome measures to better evaluate the effectiveness and appropriateness of pharmacologic pain management strategies in end-of-life care. Additionally, qualitative studies examining prescriber decision-making processes are warranted to understand the complex interplay of clinical judgment, regulatory pressures, and patient needs.

Despite these limitations, the study also possesses notable strengths. It utilizes a large, population-based sample drawn from the SEER-Medicare database, ensuring broad generalizability to older lung cancer patients across the USA. The comprehensive linkage of cancer registry data with Medicare claims enables a detailed examination of patient demographics, comorbidities, and treatment patterns. As the first study to examine the impact of hydrocodone rescheduling in end of life lung cancer patients, our findings shed light on the complex interplay between hydrocodone rescheduling and pain management practices in end-of-life lung cancer care.

## Conclusion

The policy change did not produce an immediate, significant reduction in opioid prescribing, however, the overall decline in opioid use over time underscores the broader impact of evolving regulatory and clinical guidelines. These results contribute to our understanding of how policy interventions may influence treatment patterns in end-of-life lung cancer patients for whom effective pain relief is highly important.

## Abbreviations

SEER: surveillance, epidemiology, and end results
MME: morphine milligram equivalents
NSAIDs: nonsteroidal anti-inflammatory drugs
